# Seasonal Influenza Epidemics and El Niños

**DOI:** 10.3389/fpubh.2015.00250

**Published:** 2015-11-10

**Authors:** Olusegun Steven Ayodele Oluwole

**Affiliations:** ^1^Neurology Unit, College of Medicine, University of Ibadan, Ibadan, Nigeria

**Keywords:** El Niño, La Niña, influenza, flu, seasonal, epidemic, pandemic, climate

## Abstract

Seasonal influenza epidemics occur annually during the winter in the northern and southern hemispheres, but timing of peaks and severity vary seasonally. Low humidity, which enhances survival and transmission of influenza virus, is the major risk factor. Both El Niño and La Niña phases of El Niño-southern oscillation (ENSO), which determine inter-annual variation of precipitation, are putative risk factors. This study was done to determine if seasonality, timing of peak, and severity of influenza epidemics are coupled to phases of ENSO. Monthly time series of positive specimens for influenza viruses and of multivariate El Niño-Southern Oscillation Index from January 2000 to August 2015 were analyzed. Seasonality, wavelet spectra, and cross-wavelet spectra analyses were performed. Of 31 countries in the dataset, 21 were in the northern hemisphere and 10 in the southern hemisphere. The highest number of influenza cases occurred in January in the northern hemisphere, but in July in the southern hemisphere, *p* < 0.0001. Seasonal influenza epidemic was coupled to El Niño, while low occurrence was coupled to La Niña. The moderate La Niña of 2010–2011 was followed by weak seasonal influenza epidemic. The influenza pandemic of 2009–2010 followed the moderate El Niño of 2009–2010, which had three peaks. Spectrograms showed time-varying periodicities of 6–48 months for ENSO, 6–24 months for influenza in the northern hemisphere, and 6–12 months for influenza in the southern hemisphere. Cross spectrograms showed time-varying periodicities at 6–36 months for ENSO and influenza in both hemispheres, *p* < 0.0001. Phase plots showed that influenza time series lagged ENSO in both hemispheres. Severity of seasonal influenza increases during El Niño, but decreases during La Niña. Coupling of seasonality, timing, and severity of influenza epidemics to the strength and waveform of ENSO indicate that forecast models of El Niño should be integrated into surveillance programs for influenza epidemics.

## 1 Introduction

Influenza-like illness, which is defined as fever ≥100.0°F or ≥37.8°C, cough and or sore throat without a known cause other than influenza (1), occurs annually in all parts of the world, but timing of peak and severity of epidemics vary seasonally (2). More than 3 million influenza-like cases occur annually, but attack rates are higher in children and the elderly (3). Although surveillance for antigenic drift or shift of influenza virus is intense, mortality still exceeds 250,000 deaths annually (3). While mortality is largely from complications like pneumonia and circulatory failure, neurological complications like seizures (4), Guillain–Barre syndrome (5), encephalopathy (6), rhabdomyolysis (7), and seizures (8) contribute greatly to disease burden.

Seasonal influenza epidemics peak during the winter in both the northern and the southern hemispheres. Low humidity during winter periods has been shown in epidemiological (9, 10) and experimental (11) studies to increase survival and transmission of influenza virus, although other factors like seasonal variations of contact rates (12), immune functions (13), and vitamin D concentrations contribute to infectivity (14). The determinants of the seasonality, and time-varying changes in severity of influenza epidemics are unknown. El Niño-southern oscillation (ENSO), which is a time-varying climate phenomenon, is the determinant of interannual changes in precipitation (15, 16). Although El Niño, the warm phase of ENSO, has been associated with influenza pandemics (17), and with seasonal influenza epidemics in Japan (18), time series of ENSO have not been shown to have spectra coherence with influenza-like illness in the northern and southern hemispheres, where seasonality of influenza occurrence is strongest. The putative association of La Niña, the cool phase of ENSO with seasonal influenza epidemics (19), strengthens the need for time-varying spectral analysis. This study was done to determine if seasonality, timing of peak, and severity of influenza epidemics are coupled to phases of ENSO.

## 2 Materials and Methods

The concept of this study was developed to analyze the monthly time series of ENSO indices and influenza to determine if they covary. Time series of positive specimens for influenza virus from January 2000 to August 2015 were obtained from the database of the WHO Global Influenza Programme (20).

### 2.1 ENSO Data

Multivariate El Niño-southern Oscillation Index (MEI) data were downloaded from the website of National Oceanic and Atmospheric Administration (21). The data were computed from sea-level pressure, zonal and meridional components of the surface wind, sea surface temperature, surface air temperature, and total cloudiness fraction of the sky of the South Pacific Ocean (22). Ranks of El Niño were downloaded from website of National Oceanic and Atmospheric Administration (23).

### 2.2 Time Domain Analysis

The time series of influenza, which were recorded weekly, were aggregated to monthly values. Monthly time series were fitted to ENSO and influenza data of countries, which were grouped into northern and southern hemisphere countries. All analyses were performed for each country, but only those of six countries: USA, UK, and Japan in the northern hemisphere and Argentina, South Africa, and Australia in the southern hemisphere, were displayed. Aggregated values for countries in the northern and southern hemispheres were analyzed. Autocorrelation and partial autocorrelation tests, and lag plots were done to exclude white noise and to inspect for seasonality. Stationarity was assessed using the unit root test.

Seasonality of influenza epidemics was determined by rescaling annual values to 0–1, and plotting line graphs for both northern and southern hemispheres, and for USA, UK, Japan, Argentina, South Africa, and Australia. Radial plots were drawn to show the distribution of cases by months of the year for both hemispheres. Rayleigh’s test of uniformity was performed to determine if the distributions of influenza were uniform all year in each hemisphere, while Watson’s two-sample test of homogeneity was performed to determine if the distributions of influenza in the northern and southern hemispheres were statistically different.

The waveform of ENSO of 2009–2010, during which the influenza pandemic of 2009–2010 occurred, was compared with that of 2002–2003, which was moderate in strength, with ENSO of 2004–2005 and 2006–2007, which were weak, and with ENSO of 1982–1983 and 1997–1998, which were very strong.

### 2.3 Wavelet Spectral Analysis

Since the ENSO is quasiperiodic, spectral analysis with fast Fourier transform method, which only detects frequencies, but not their temporal changes, was considered inadequate. The time series were analyzed using wavelet spectra analysis, which shows time-varying frequencies. The methodology of wavelet spectral methods has been described for epidemiological (24, 25), human (26), and environmental data (27, 28).

The time series were transformed to time-frequency domain using Morlet wavelet, which was defined as (24, 25)

$$\psi_{0}\left(\eta \right)=\pi^{-1∕4}e^{i\omega_{0}\eta }e^{-\eta^{2}∕2}$$

where ω_0_ is dimensionless frequency and η is dimensionless time. The continuous wavelet transform of time series (*x_n_*, *n* = 1, …,N)

$$W_{n}^{x}\left(s\right)=\sqrt{\frac{\delta_{t}}{s}}\overset{N}{\underset{n^{\prime }=1}{\sum }}\;x_{n}^{\prime }\psi_{0}\left[\left(n^{\prime }-n\right)\frac{\delta_{t}}{s}\right]$$

with uniform time steps δ_t_, was defined as the convolution of *x_n_* with the scaled and normalized wavelet (24, 25). To determine the statistical relationship of two time series the cross wavelet transform (24, 25), which was based on the equation

$$D\left(\frac{\left|W_{n}^{x}\left(s\right)W_{n}^{y}⋆\left(s\right)\right|}{\sigma_{x}\sigma_{y}}<p\right)=\frac{Z_{v}\left(p\right)}{v}\sqrt{P_{k}^{x}P_{k}^{y}}$$

where *Z_v_*(*p*) was the confidence level associated with probability *p*, and $P_{k}^{x}$ and $P_{k}^{y}$ were the power spectra, and the wavelet coherency phase (24, 25) shown as

$$R_{n}^{2}\left(s\right)=\frac{|S\left(s^{-1}W_{n}^{\text{xy}}\left(s\right)\right){|}^{2}}{S\left(s^{-1}|W_{n}^{x}\left(s\right){|}^{2}\right)\cdot S\left(s^{-1}|W_{n}^{y}\left(s\right){|}^{2}\right)}$$

were done.

The global wavelet spectra, the equivalent of the Fourier power spectrum smoothed by the Morlet wavelet function (24, 29), shown below

$${\bar{W}}^{2}\left(s\right)=\frac{1}{N}\overset{N-1}{\underset{n=0}{\sum }}\;{|W}_{n}(s){|}^{2}$$

was done to show all the frequencies in the spectra of each time series. Phase plots were drawn to illustrate the phase shift between the time series.

### 2.4 Statistical Programs

Statistical analyses were performed using the R Statistical Programming and Environment, Austria, version 3.2.1 (30). Specifically, wavelet analyses were performed using the biwavelet package, phase plots using the WaveletComp package, graphics using the ggplot2 package, plotrix package for radial plots, and circular package for direction statistics.

## 3 Results

**Figure 1:**
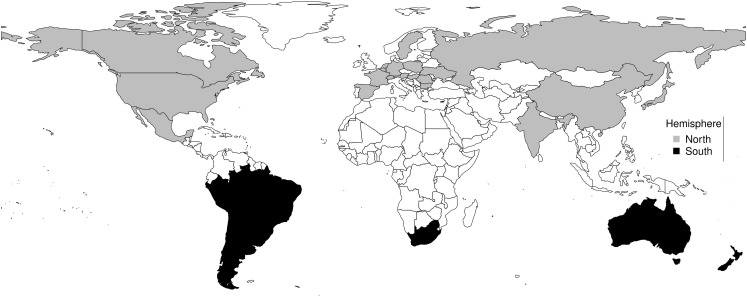
**Map of northern and southern hemisphere countries**.

There were 31 countries in the dataset, 21 in northern hemisphere and 10 in southern hemisphere (Figure 1). Countries in the northern hemisphere were Austria, Belgium, Bulgaria, Canada, China, France, Germany, Hungary, India, Japan, Mexico, Netherlands, Norway, Poland, Romania, Russia, Spain, Sweden, Switzerland, United Kingdom, Ukraine, and the United States, while those in the southern hemisphere were Argentina, Australia, Bolivia, Brazil, Chile, New Zealand, Paraguay, Peru, South Africa, and Uruguay.

**Figure 2:**
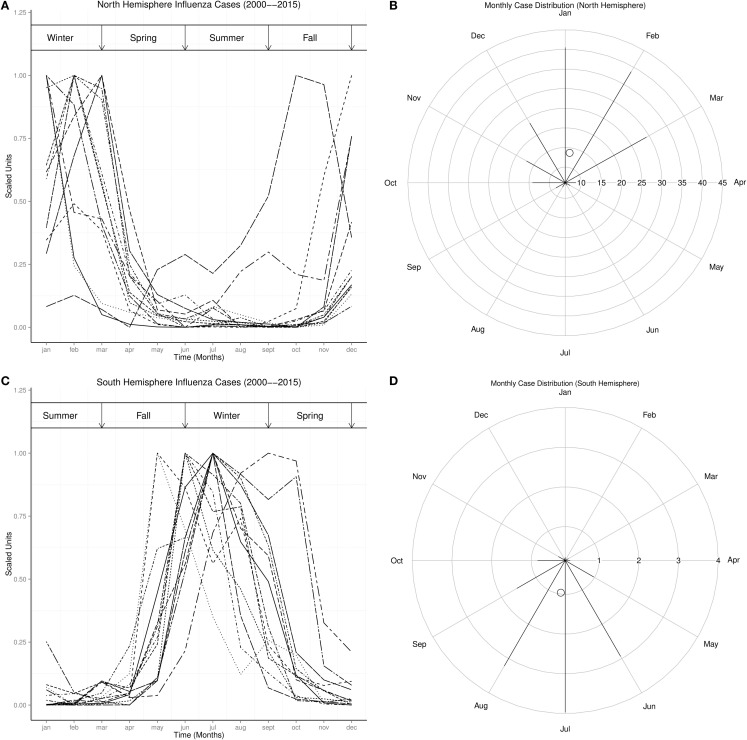
**Seasonal Distribution of Influenza Wt is Winter, Sp is Spring, Sm is Summer, and Fa is Fall**. **(A)** Monthly plots of time series of influenza cases in north hemisphere. **(B)** Radial plot of influenza cases in north hemisphere. **(C)** Monthly plots of time series of influenza cases in south hemisphere. **(D)** Radial plot of influenza cases in north hemisphere.

**Figure 3:**
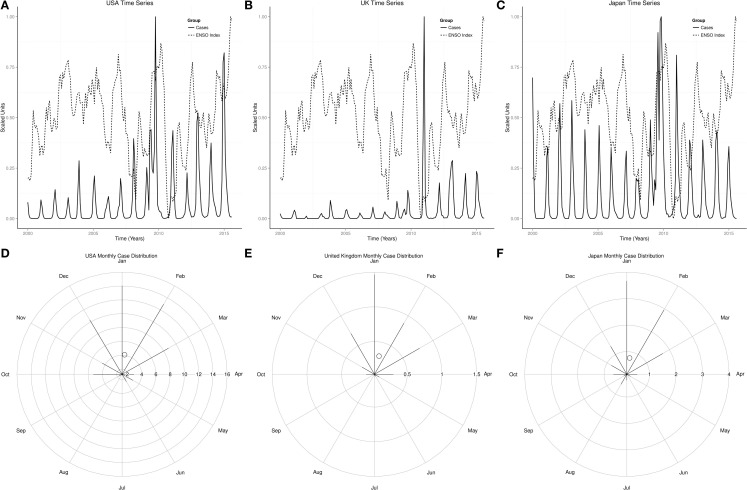
**Time series and monthly distribution of influenza USA, UK, and Japan**. **(A)** Time series of influenza cases in the USA. **(B)** Time series of influenza cases in the UK. **(C)** Time series of influenza cases in Japan. **(D)** Radial plot of influenza cases in the USA. **(E)** Radial plot of influenza cases in the UK. **(F)** Radial plot of influenza cases in Japan.

**Figure 4:**
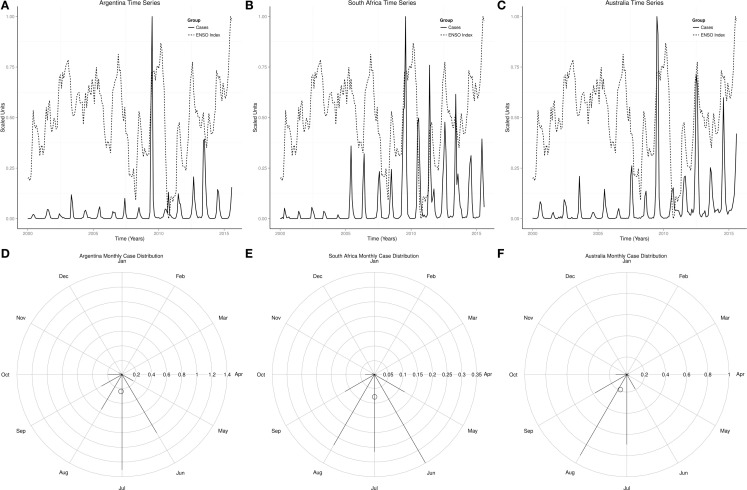
**Time series and monthly distribution of influenza in Argentina, South Africa, and Japan**. **(A)** Time series of influenza cases in Argentina. **(B)** Time series of influenza cases in South Africa. **(C)** Time series of influenza cases in Australia. **(D)** Radial plot of influenza cases in Argentina. **(E)** Radial plot of influenza cases in South Africa. **(F)** Radial plot of influenza cases in Australia.

Figures 2A,C showed that influenza peaked predominantly in the winter of both northern and southern hemispheres, but infrequently in the fall. The radial plot showed that high number of influenza cases occurred in the northern hemisphere from December to March, but was highest in January, while in the southern hemisphere high number of influenza cases occurred from June to August, but highest in July Figures 2B,D. The centroid was in January in the northern hemisphere, but in July in the southern hemisphere (Figures 2B,D). Rayleigh’s test of uniformity showed that influenza occurrence was not uniform all year in both northern and southern hemispheres, *p* < 0.0001. Watson’s two-sample test of homogeneity showed statistically significant difference between the monthly distribution of influenza in the northern and southern hemispheres, *p* < 0.0001. The highest number of influenza cases also occurred in January in USA, UK, and Japan, but in July in Argentina, June in South Africa, and August in Australia (Figures 3D–F and 4D–F).

**Figure 5:**
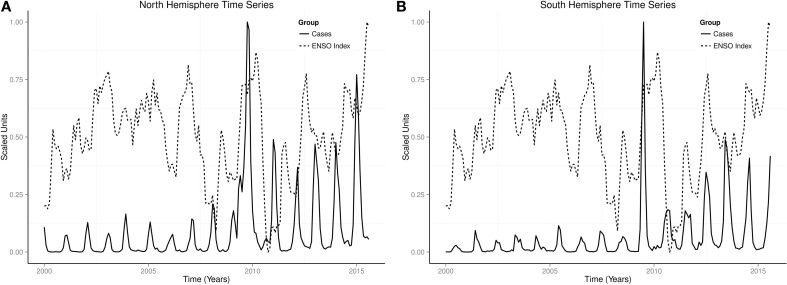
**Time series of influenza in northern and southern hemispheres**. **(A)** Time series of influenza cases in north hemisphere. **(B)** Time series of influenza cases in south hemiphere.

Monthly time series of influenza and indices of ENSO showed rhythmic changes in the northern and southern hemispheres (Figures 5A,B). Monthly time series of influenza and indices of ENSO in USA, UK, Japan, Argentina, South Africa, and Australia also showed rhythmic changes (Figures 3A–C and 4A–C). Rhythmic changes of ENSO and influenza are coupled in both hemispheres, and in all countries in each hemisphere.

**Figure 6:**
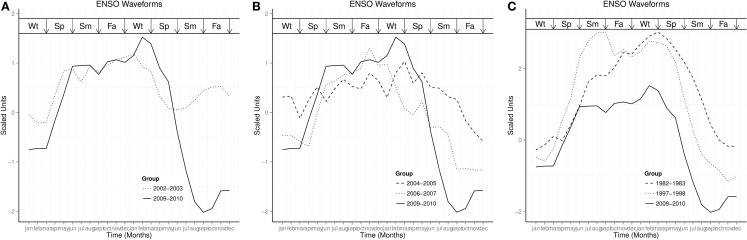
**Waveforms of ENSO Wt is Winter, Sp is Spring, Sm is Summer, and Fa is Fall**. **(A)** Comparison of 2009–2010 ENSO with moderate ENSO. **(B)** Comparison of 2009–2010 ENSO with weak ENSO. **(C)** Comparison of 2009–2010 ENSO with very strong ENSO.

Four El Niños occurred between 2000 and 2014, while one is currently developing in 2015. Comparisons of the waveform of El Niño of 2009–2010, during which influenza pandemic occurred, with the El Niño of 2002–2003, which was moderate, with El Niños of 2004–2006 and 2006–2007, which were weak, and with El Niños of 1982–1983 and 1997–1998, which were very strong, are shown in Figures 6A–C. The rising phase of the ENSO waveform was typical in spring, the peak phase from spring to spring, and decay phase from spring to summer. The waveform of ENSO of 2009–2010 had higher and longer duration peak than the moderate El Niño of 2002–2003 and weak El Niños of 2004–2005 and 2006–2007, Figures 6A,B, but lower and shorter duration peak than the El Niños of 1982–1983 and 1997–1998 (Figure 6C).

**Figure 7:**
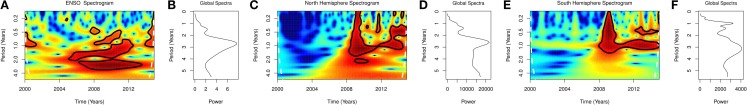
**Spectral of influenza-like illness and ENSO**. **(A)** Spectrogram of ENSO. **(B)** Global spectra of ENSO. **(C)** Spectrogram of influenza cases in north hemisphere. **(D)** Global spectra of influenza cases in north hemisphere. **(E)** Spectrogram of influenza cases in south hemisphere. **(F)** Global spectra of influenza cases in south hemisphere.

Global wavelet spectra showed peaks at 18 and 32 months for ENSO, at 12, 18, and 32 months for northern hemisphere influenza, and at 6, 12, and 24 months for southern hemisphere influenza (Figures 7B,D,F). Spectrograms showed time-varying periodicities of 6–48 months for ENSO, 6–24 months for northern hemisphere influenza, and 6–12 months for southern hemisphere influenza (Figures 7A,C,E).

**Figure 8:**
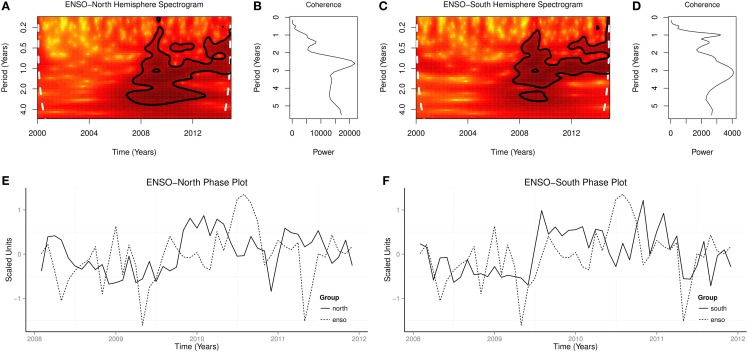
**Cross spectra of influenza-like illness and ENSO**. **(A)** Cross spectrogram of ENSO and influenza cases in north hemisphere. **(B)** Coherence squared of ENSO and influenza cases in north hemisphere. **(C)** Cross spectrogram of ENSO and influenza cases in south hemisphere. **(D)** Coherence squared of ENSO and influenza cases in south hemisphere. **(E)** Phase plot of ENSO and influenza cases in north hemisphere. **(F)** Phase plot of ENSO and influenza cases in south hemisphere.

Coherence squared showed peaks at 12, 18, and 32 months for ENSO and northern hemisphere influenza cases, and at 6, 12, 18 months for ENSO and southern hemisphere (Figures 8C,D). Cross spectrograms showed time-varying periodicities at 6–36 months for ENSO and influenza in both hemispheres, *p* < 0.0001 (Figures 8A,B). Phase plots showed that time series of influenza lagged ENSO in both hemispheres (Figures 8E,F).

## 4 Discussion

Influenza occurs all year in the northern and southern hemispheres, but seasonal epidemics occur predominantly during the winter in both hemispheres, which is from December to February in the northern hemisphere, but from June to August in the southern hemisphere (Figures 1 and 2). The monthly distribution of influenza in the USA, UK, and Japan, which show highest occurrence in January, but in July in Argentina, June in South Africa, and August in Australia, illustrates the geospatial differences in timing of influenza epidemics Figures 3 and 4. Timing of highest occurrence of influenza from December to February in the northern hemisphere, and from June to August in the southern hemisphere, indicates the calendar difference in occurrence between the two hemispheres. Although the calendar timing is statistically different between the northern and southern hemispheres, there is no seasonal difference since influenza epidemics occur in the winter of each hemisphere. Thus, strong seasonality of influenza epidemics is attributable to environmental factors, but not only to changes in virulence of influenza viruses.

Occurrence of influenza show rhythmic changes in both northern and southern hemispheres (Figures 2–4). Influenza epidemics, which lag El Niño phase of the ENSO, and low influenza occurrence, which lag La Niña, indicate that influenza time series covary with ENSO in both hemispheres (Figures 2–4). Although every ENSO has peaks and troughs, which impact on climate annually, El Niños do not occur during every ENSO. El Niño, which is strongly coupled to season cycles (31), occurs when running 3-month mean sea surface temperature anomaly exceeds baseline threshold, defined by Oceanic Nino Index as ≥0.5°C increase in temperature in the Niño 3.4 region (5°N–5°S, 120°–170°W) of the Pacific Ocean for five consecutive periods (32). La Niña occurs when the anomaly is ≤−0.5°C (32). Figure 6, which shows that the rising phase of ENSO is in spring, peaks from spring to spring, and decay phase from spring to summer, illustrates the strong coupling of ENSO to season cycles. Figure 6 also shows that the peak phase last several seasons, but with different amplitudes, unlike the rising and decay phases, which last one or two seasons. The ENSO, therefore, varies not only in amplitude and duration (33) but also in waveform. The timing of the peaks of the ENSO, therefore, coincides with the winter of both hemispheres when seasonal influenza epidemics occur. Thus, maximal strength of ENSO precedes seasonal influenza epidemics in both hemispheres.

ENSO varies on multiple timescales, which include annual, decadal, and multidecadal timescales (15, 16). The periodicities of ENSO were 3–4 years between 1872 and 1910, 5–7 years between 1911 and 1960, and 5 years between 1970 and 1972 (25, 34). Global wavelet spectra of ENSO between 2000 and 2015, which show peaks at 1.5 and 2.5 years, show the presence of rhythms in the short timescale (Figures 7B,D,F). The global spectra of influenza epidemics, which show peaks at 1.0, 1.5, and 2.5 in the northern hemisphere and 0.5, 1.0, and 2.0 in the southern hemisphere, also show the presence of rhythms in short timescale (Figures 7B,D,F). Thus, similar rhythms are present in the spectra of ENSO and influenza.

Time-varying periodicities in the spectrograms of ENSO have shown the irregular occurrence of El Niños (25). It has been proposed that the irregularity of ENSO is due to several factors that include chaos, Madden–Julian oscillation, PDO, and global warming (35). (Figures 7A,C,E). Highly significant coherence of ENSO and influenza at 1.0, 1.5, and 2.5 years indicate that the two time series are not independent, but the phase lag shows that ENSO rhythms lead influenza rhythms (Figures 8C–F). Cross spectrograms show, however, that highly statistically significant time-varying coherence are present from periodicities of 0.5–3.0 years during seasonal influenza epidemics and influenza pandemic of 2009–2010 (Figures 8A,B). Thus, there is biological plausibility that the environmental impact of changes induced by ENSO contributes to the causation of seasonal epidemics and pandemic of influenza.

Severity of seasonal influenza epidemics vary from mild to severe (36, 37), while the strength of El Niño is graded from weak to very strong (22, 38). In the USA, the influenza season was moderately severe in 2003–2004 and 2007–2008, moderate in 2002–2003, but mild in 2011–2012 (2). The El Niños of 2004–2005 and 2006–2007 were weak, moderate in 2002–2003, but moderate La Niña occurred in 2010–2011. The moderate and moderately severe seasons, therefore, followed El Niño years, while the mild influenza season followed La Niña. Since the virulent H3N2 influenza virus circulated during the moderately severe seasons of 2003–2004 and 2007–2008 and the mild season of 2011–2012, the virulence of the virus alone is not sufficient to induce severe epidemic. The weak El Niños of 2003–2004 and 2007–2008 that had H3N2 influenza virus in circulation were moderately severe, while the moderate El Niño of 2002–2003, which had B/H1N1 viruses in circulation, was moderate. Thus, El Niño accentuates severity of seasonal epidemics while La Niña attenuates it. Currently, El Niño of 2015, which is gaining strength, is lagged by rising occurrence of influenza in the southern hemisphere (Figures 3A–C and 5B). If the observed rhythmic pattern continues, the 2015–2016 influenza season is expected to be severe.

In contrast to seasonal influenza epidemics, four influenza pandemics have occurred since 1900, in 1918–1919, 1957–1958, 1968–1969 (39), and 2009–2010. All influenza pandemics peaked two to three times, unlike the single peaks of seasonal influenza epidemics. Influenza pandemics from 1580–2013 have been observed to lag El Niños by 0–2 years (17). The 2009–2010 influenza pandemic, which had three peaks, followed the moderate El Niño of 2009–2010, which also had three peaks. The cross spectrogram of ENSO and influenza, which had high coherence during the period of the pandemic indicates that environmental factors also contribute to occurrence of influenza pandemic. It has been observed that influenza pandemics did not occur during the very strong El Niños of 1982–1983 and 1997–1998 (22, 38, 40), which were stronger than the El Niño of 2009–2010. Since the strength of El Niño correlates with its impact on climate, the absence of influenza pandemics following very strong El Niños suggests that the influenza virus requires optimal climate for transmission. An experimental study, which showed that transmission of influenza virus in guinea pig model was optimal at relative humidity of 20–35% (11) supports this speculation. It is, however, noteworthy that the El Niños of 2002–2003 and 2009–2010 were both moderate, but the influenza epidemic season of 2002–2003 was moderate. The circulating influenza virus was pH1N1 in 2009–2010, but B/H1N1 influenza viruses circulated in 2002–2003. The basis for the seasonality of influenza has been extensively reviewed (41), but the interaction of climate with influenza virus to induce antigenic drift or shift, and with the host to increase susceptibility remains unclear. Although the influenza pandemic waves of 2009–2010 were also coupled to ENSO, the putative mechanism of the interaction is unclear.

Although vaccination is the most effective method of protection against influenza virus (3), the effectiveness of vaccination is still very low in some influenza seasons. The effectiveness of vaccination against seasonal influenza in the USA from 2000–2014, which ranged from 10–60%, but was 23% in 2014–2015 influenza season (42), shows that vaccination fails to protect a large proportion of the population. Incorporation of forecasts of El Niño should improve preparedness for severe influenza epidemic seasons.

## 5 Limitations of Study

A major limitation of the study is the lack of time series of influenza for previous influenza pandemics, which precludes similar analysis. There is, however, the need to use different methodology to further study the relationship of influenza pandemics and ENSO.

## 6 Conclusion

Seasonal (43, 44) and geospatial (45) differences in occurrence of several infectious diseases have been linked to El Niño. Seasonal epidemics of meningococcal meningitis in the Sahel, Central, and southern Africa (46), dengue in Thailand (47), cholera in Bangladesh (48, 49), malaria in Venezuela (50), and rodent-borne zoonosis in China (51), have been linked to El Niño activity. Konzo, a spastic neurological syndrome that occurs in epidemics during severe droughts and food shortages in East, Central, and Southern Africa, has also been attributed to El Niño activity (52). Thus, the impact of El Niño on precipitation of different parts of the Earth through teleconnections promotes epidemics of infectious diseases and of toxico-nutritional disease.

ENSO and influenza are highly coupled to season cycles, but influenza epidemics occur during the winter of northern hemisphere, which is from December to February, and during the winter of southern hemisphere, which is from June to August. The ENSO peaks from spring to spring in the northern hemisphere, which includes the winter of both hemispheres. Severity of seasonal influenza epidemics increases during El Niño, but decreases during La Niña. Highly significant spectra coherence of time series of ENSO and influenza during seasonal epidemics and the pandemic of 2009–2010 indicate that the impact of climate variability contributes to occurrence of both. The currently rising occurrence of influenza in the southern hemisphere, which lags the rising phase of 2015 El Niño indicate that the 2015–2016 influenza season will be severe. Coupling of seasonality, timing, and severity of influenza epidemics to the strength and waveform of ENSO indicate that forecast models of El Niño should be integrated into surveillance programs for influenza.

## Conflict of Interest Statement

The author declares that the research was conducted in the absence of any commercial or financial relationships that could be construed as a potential conflict of interest.
